# Sardinians Genetic Background Explained by Runs of Homozygosity and Genomic Regions under Positive Selection

**DOI:** 10.1371/journal.pone.0091237

**Published:** 2014-03-20

**Authors:** Cornelia Di Gaetano, Giovanni Fiorito, Maria Francesca Ortu, Fabio Rosa, Simonetta Guarrera, Barbara Pardini, Daniele Cusi, Francesca Frau, Cristina Barlassina, Chiara Troffa, Giuseppe Argiolas, Roberta Zaninello, Giovanni Fresu, Nicola Glorioso, Alberto Piazza, Giuseppe Matullo

**Affiliations:** 1 Department of Medical Sciences, University of Turin, Turin, Italy; 2 HuGeF Human Genetics Foundation, Turin, Italy; 3 Hypertension and Related Diseases Center, AOU, University of Sassari, Sassari, Italy; 4 Department of Health Sciences, University of Milan, Milan, Italy; 5 Filarete Foundation, Genomic and Bioinformatics Unit, Viale Ortles 22/4, Milan, Italy; Estonian Biocentre and Tartu University, Estonia

## Abstract

The peculiar position of Sardinia in the Mediterranean sea has rendered its population an interesting biogeographical isolate. The aim of this study was to investigate the genetic population structure, as well as to estimate Runs of Homozygosity and regions under positive selection, using about 1.2 million single nucleotide polymorphisms genotyped in 1077 Sardinian individuals. Using four different methods - fixation index, inflation factor, principal component analysis and ancestry estimation - we were able to highlight, as expected for a genetic isolate, the high internal homogeneity of the island. Sardinians showed a higher percentage of genome covered by RoHs>0.5 Mb (F_RoH%0.5_) when compared to peninsular Italians, with the only exception of the area surrounding Alghero. We furthermore identified 9 genomic regions showing signs of positive selection and, we re-captured many previously inferred signals. Other regions harbor novel candidate genes for positive selection, like *TMEM252*, or regions containing long non coding RNA. With the present study we confirmed the high genetic homogeneity of Sardinia that may be explained by the shared ancestry combined with the action of evolutionary forces.

## Introduction

Due to the geographic isolation of Sardinia in the Mediterranean sea, Sardinian population can be considered a genetic isolate. Faunal and floral endemism underline this peculiarity, which is reflected also in the genetic and cultural structure of the human population. For such reasons, Sardinians have been object of numerous investigations in the fields of anthropology and population genetics [1], [2], [3], [4].

Several studies have shown that the genome of current Sardinia inhabitants still contains some signatures of a long history of isolation. These features make this genetic isolate an ideal population for association studies [5], [6], [7]. However, much remains to be discovered about the genomic regions that were inherited from common ancestors, such as the short Runs of Homozygosity (RoHs), or the portions of the genome that have been selected by positive sweep.

In the present study, we have analyzed the genetic structure of the Sardinian population by using 1.2 million single nucleotide polymorphisms (SNPs) from 1077 Sardinians previously included in a genome-wide association study (GWAS) [8], and 79 healthy individuals from peninsular Italy. The aims of the study were the following: (i) reconfirming, through the use of autosomal genome wide data, the homogeneity of Sardinia population at the inter-regional level; (ii) inferring, through the use of RoHs, the population genetic history by estimating the background level of shared ancestry within the island and by comparing it with peninsular Italy; (iii) identifying signals of positive selection.

## Materials and Methods

### Data sets

**Figure 1:**
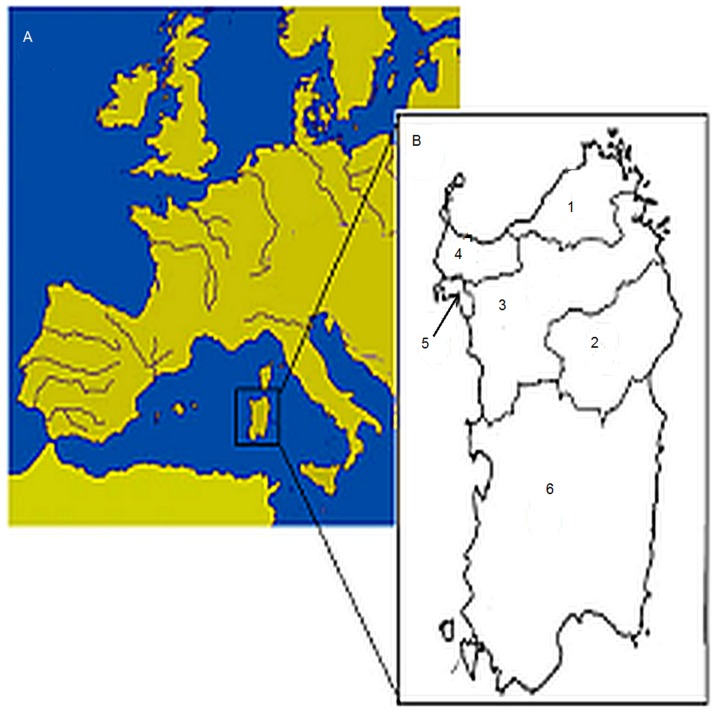
Map of Mediterranean basin showing the localization of Sardinia and Sardinian linguistic domains. A) Map of the Mediterranean basin showing the geographic position of Sardinia. B) The Sardinian linguistic domains: 1 = *Gallurese* (77 individuals); 2 = *Nuorese* (88); 3 = *Logudorese* (385); 4 = *Sassarese* (342); 5 = *Alghero* (87); 6 = *Campidanese* (98).

Genotypic data from 1077 healthy subjects from Sardinia were used as primary data-set. Those samples were collected in the frame of an international consortium for GWAS on hypertension (HyperGene) and described elsewhere [8]. Subjects were clustered according to birth place, dividing Sardinia on the basis of the language spoken as suggested by Contini and coworkers [9], [10]. In the present work, a simplification of this approach was used by dividing the island into six main macro-areas as displayed in Figure 1: *Gallurese* (n = 77), *Nuorese* (n = 88), *Logudorese* (n = 385), *Sassarese* (n = 342), *Alghero* (n = 87) and *Campidanese* (n = 98). Part of the samples (n = 250) have been already analyzed in a previous work [11].

An additional group consisting of 79 Italian individuals was included in the study to perform a comparison of Sardinian genetic background with the Italian mainland. The peninsular Italian subjects were genotyped in our laboratory for more than 1M SNPs (HumanOmni1–QUAD v1.0 BeadChip, Illumina Inc, S. Diego, CA, USA). To compare Sardinia and Italy, only SNPs common to both data-sets were considered (∼520 k markers).

All samples were collected with informed consent and analyzed anonymously. Their use for population genetics studies was approved by the ethics committee of the Human Genetics Foundation (HuGeF) in Turin.

### Quality Assessment and Control Procedure

Stringent quality control procedures were applied when performing SNPs genotyping analysis. Samples with an individual call rate lower than 98% were excluded. SNPs with minor allele frequency (MAF) less than 0.01 were excluded, as well as those who failed the Hardy-Weinberg equilibrium test (p<1×10^−3^). In order to estimate individual number of RoHs, SNP markers on sex chromosomes were excluded. After quality control procedures, the Sardinian data-set contained a total of 946,970 SNPs.

### Statistical Data Analyses

Analysis was performed at different levels. The first one was to assess the genetic structure within Sardinia. A second level was aimed at reconstructing the genetic population history through RoHs analysis, and the identification of genomic regions under positive selection.

### Sardinian population structure

**Figure 2:**
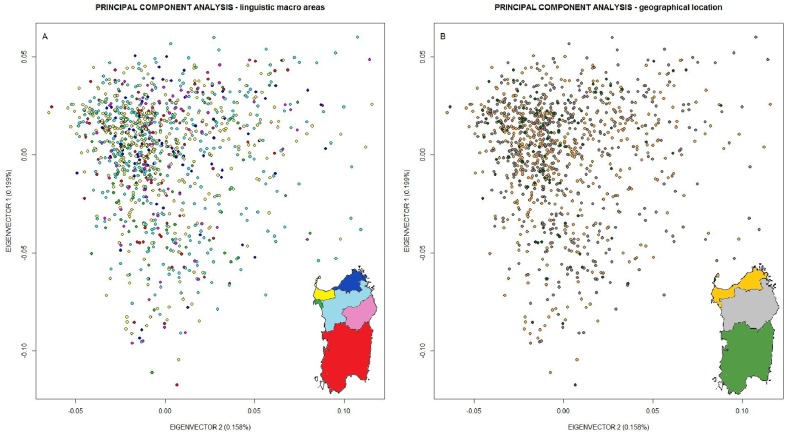
SNP-Based Principal Component Analysis of 1,077 individuals from Sardinia. Figure 2 A) division accounting linguistic macro-areas. Key of the colors: red: *Campidanese*; green: *Alghero*; deep blue: *Gallurese*; light blue: *Logudorese*; yellow: *Sassarese*; purple: *Nuorese*. Figure 2 B) division accounting geographical areas. Key of the colors: green: *Southern Sardinia*; grey: *Central Sardinia*; yellow: *Northern Sardinia*.

Principal Component Analysis (PCA) was performed using the complete set of markers, with the algorithm implemented in the R package [12] SNPRelate [13]. The PCA values of each individual sample have been plotted on the space defined by the first 2 eigenvectors: subjects from the same linguistic macro-area or the same geographic area have been displayed with identical color (Figure 2A and B).

We used the first four principal components (PCs) as predictors in a multinomial logistic regression using the linguistic macro-area as dependent outcome. We then evaluated the prediction accuracy of the described model: for each sample the most probable linguistic macro-area estimated by the model was compared to the real one (10,000 iterations).

Pairwise inflation factors (λ_GC_) [14] between the six macro-areas were computed through PLINK software [15], simulating a case-control study between each pair of macro-areas (*–adjust* option).

We used two different methods to calculate F_st_: the first one was roundly intended to produce estimates on data with significant inbreeding (like the six macro-areas) while the second one was meant for panmictic populations (Sardinia versus peninsular Italy). Pairwise genetic F_st_ correct for inbreeding between the six macro-areas was estimated as suggested in Reich *et al.* [16]. F_st_ between the Sardinian and peninsular Italians populations was estimated using the Hudson estimator for genome-wide data [17], as suggested in Bhatia *et al.* [18]. The R code to compute both estimators is available in Text S1.

Mean inbreeding coefficients were estimated on the basis of the observed versus expected number of homozygous genotypes over the whole genome, using the data set containing also peninsular Italian individuals (PLINK software (*–het* option)). Differences between Sardinian population and peninsular Italians were evaluated using a T test. The software ADMIXTURE [19] was used to estimate the ancestry for each individual in Sardinian population and in peninsular Italian subjects. A cross validation error-based method was applied to detect the number of clusters (K) after 20 runs.

### Runs of Homozygosity Analysis

RoHs were estimated separately for Sardinians and peninsular Italians (PLINK software (*–homozyg* option)). The following parameters were used for the estimation algorithm:1) a sliding window of 5000 kb, with a minimum of 50 SNPs that must be present in the region considered; 2) for a given window, a maximum of one heterozygous and a maximum of five missing calls allowed; 3) each SNP was considered to be part of an homozygous segment when the proportion of homozygous windows overlapping that position was above the threshold value of 0.05.

We identified 6 RoH categories based on the length of the genomic region of homozygosity (0.5–1 Mb,1–2 Mb, 2–4 Mb, 4–8 Mb, 8–16 Mb, >16 Mb), and estimated the proportion of individuals with RoHs of different size in each Sardinia's macro-areas. Differences between Sardinian macro-areas and peninsular Italy were evaluated using a T test. We also estimated the proportion of the genome covered by regions of homozygosity (F_RoH_%) according to McQuillian *et al.* [20]. Two classes of RoHs were considered in this analysis: RoH≥0.5 Mb, and RoH≥5 Mb. For each class and for each macro-area we computed the average F_RoH_% over all individuals, as well as the average sum of length of all RoHs in the same class. A T test was performed to evaluate the differences between the two classes of RoH within each macro-area and Italy.

### Extended haplotype homozygosity (EHH) and related tests

FastPHASE software [21] was used to perform a haplotype phase estimation. The estimated haplotypes were subsequently used to detect footprints of selection from haplotype structure.

For each SNPs we computed the EHH statistic [22] of both alleles (ancestral and derived), as well as the integrated haplotype score (iHS) [23]. The algorithm is implemented in the R package *rehh* [24]. For this specific analysis we employed a total of ∼900 k markers for which information about ancestral allele was available in the public databases [25]. Lastly, we searched for chromosomal regions that showed enrichment of SNPs with |iHS|>4, using the approach suggested by Voight *et al.* [23]. Permutation based correction for multiple comparisons was applied.

## Results

**Figure 3:**
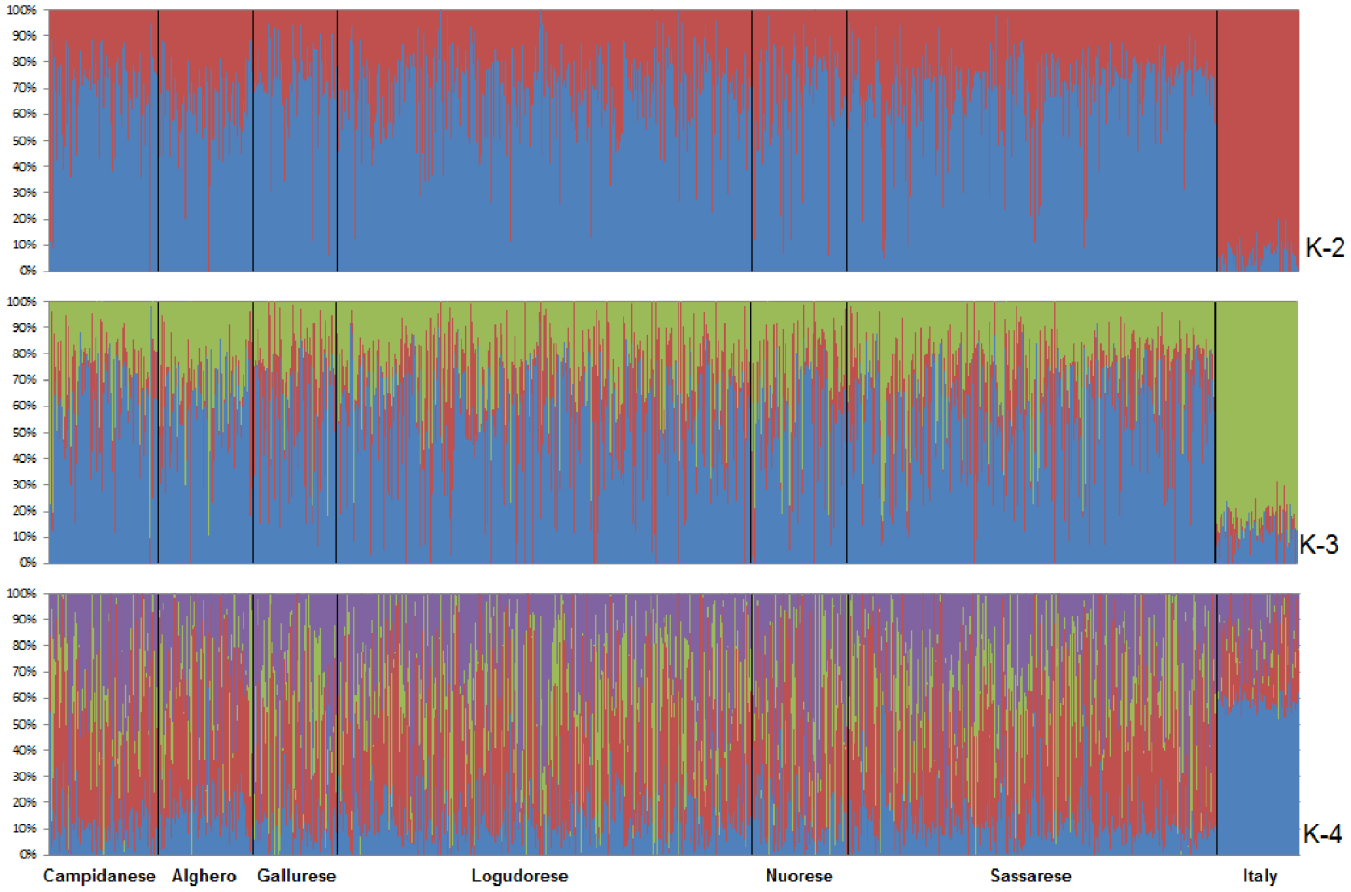
ADMIXTURE software results for K = 2–4. Ancestry for each individual inferred using ADMIXTURE software.

The multinomial logistic regression model using the first four eigenvector as predictors of the linguistic macro-areas showed very low accuracy (from a minimum of 0.2044 to a maximum of 0.3201, 10,000 iterations), suggesting a high degree of homogeneity within Sardinian population. No sub-populations were apparently identified projecting the Sardinian samples onto a two-dimensional space (based on the first two eigenvectors) using all autosomal markers (934,288 SNPs) within the linguistic macro-areas (Figure 2A), or dividing the island in 3 geographic regions (Figure 2B). The distribution of the first four eigenvectors is shown in Figure S1. All pairwise F_st_ values inbreeding corrected within Sardinian linguistic macro-areas were close to zero (Table 1), and we observed a F_st_ estimator of 0.003 (p-value<0.0001 95% C.I. 0.0025–0.0033) when comparing Sardinia to peninsular Italy. Pairwise inflation factors (λ_GC_) were strictly close to 1 (from 1.01 to 1.05) (Table 1). The ancestry analysis highlighted a common genetic background for all the individuals of the island (Figure 3). The observed shared ancestry made unfeasible any attempt to cluster individuals on the basis of their place of birth. By using the cross validation error, we indicated “K = 2” as the number of clusters more compatible with the data. Furthermore higher values of K did not reveal additional population-specific ancestries.

**Table 1:** F_st_ values (in bold) and genomic control inflation factor (λ_GC_) (*in italics*) between Sardinian linguistic macro-areas.

λ_GC_/F_st_	Campidanese	Alghero	Gallurese	Logudorese	Nuorese	Sassarese
Campidanese	**-**	**4.4×10^−5^**	**1.1×10^−4^**	**3.2×10^−5^**	**2.2×10^−5^**	**8.5×10^−6^**
Alghero	*1.037*	***-***	**1.5×10^−4^**	**9.1×10^−5^**	**1.1×10^−4^**	**7.1×10^−5^**
Gallurese	*1.051*	*1.047*	**-**	**1.2×10^−4^**	**1.4×10^−4^**	**1.1×10^−4^**
Logudorese	*1.018*	*1.041*	*1.040*	**-**	**6.1×10^−5^**	**4.2×10^−5^**
Nuorese	*1.019*	*1.027*	*1.040*	*1.028*	**-**	**4.0×10^−5^**
Sassarese	*1.012*	*1.032*	*1.046*	*1.046*	*1.021*	**-**

The percentage of genome covered by RoHs>0.5 Mb (F_RoH_%_0.5_) was higher in Sardinians when compared to peninsular Italians, with the only exception of the area surrounding Alghero (Table 2). No significant difference was observed between Sardinians and Italians when comparing the fraction of the genome covered by RoHs>5 Mb (F_RoH_%_5_) (Table 2).

**Table 2:** Mean genomic inbreeding coefficients (F_RoH_ %) using 0.5 and 5 Mb minimum RoH thresholds and mean sum of RoH.

	F_RoH_%≥0.5	F_RoH_%≥5	Mean (SD) sum of RoH (Mb)			
			mean≥0.5		mean≥5	
Campidanese	3.08*	0.49	82.55*	3.81	13.24	3.14
Alghero	2.71	0.29	72.77	2.86	7.86	2.28
Gallurese	3.10*	0.51	83.09*	4.39	13.65	3.82
Logudorese	2.96*	0.41	79.33*	1.79	11.06	1.53
Nuorese	2.89*	0.42	77.61*	3.74	11.15	3.26
Sassarese	2.94*	0.44	78.84*	2.05	11.67	1.84
Italy	2.52	0.47	67.55	4.98	12.64	4.28

* *p-value* smaller than 0.05 when comparing each linguistic macro-area to peninsular Italy.

Significant differences were observed in the mean inbreeding coefficients between Campidanese, Gallurese, Sassarese, and Logudorese macro-areas and peninsular Italy (Table 3).

**Table 3:** Mean inbreeding coefficients.

	Mean inbreeding coefficient	SE	*p-Value*
Campidanese	0.0106	0.00015	0.002
Alghero	0.0058	0.00014	0.26
Gallurese	0.0100	0.00022	0.01
Logudorese	0.0086	0.00002	0.003
Nuorese	0.0079	0.00016	0.06
Sassarese	0.0082	0.00004	0.01
Italy	0.0046	0.00001	-

Mean inbreeding coefficients, standard errors (SE) and T test *p-Values* of Sardinia macro-areas and peninsular Italy.

Since the distribution of different classes of RoH allows to study different demographic patterns involving a population, we further divided RoHs into 6 different classes, as shown in Table 4. Sardinia had a higher number of RoHs than Italy for 2 classes of RoH: 0.51 Mb, and 1–2 Mb (p-value<0.05). Regarding longer RoH classes (8–16 Mb and >16 Mb) no significant difference was found between the two regions, with the exception of Campidanese for the class 8–16 Mb. Comparing the class of RoH longer than 2 Mb, the Alghero district and Sassarese were not found statistically different from peninsular Italy.

**Table 4:** Percentage of the accessible genome occupied (2.84 Gb) and mean sum of RoH in Mb (with standard errors SE) for six classes of RoH.

	0.5–1 Mb			1–2 Mb			2–4 Mb			4–8 Mb			8–16 Mb			>16 Mb		
	% RoH	mean	SE	% RoH	mean	SE	% RoH	mean	SE	% RoH	mean	SE	% RoH	mean	SE	% RoH	mean	SE
Campidanese	1.23	32.92*	0.53	0.98	26.25*	0.56	0.31	8.27* °	0.6	0.2	5.28* °	0.78	0.17	4.66*	1.26	0.19	5.18	1.74
Alghero	1.19	31.86*	0.56	0.95	25.61*	0.58	0.26	6.89	0.51	0.1	2.66	0.57	0.13	3.46	1.06	0.09	2.29	1.12
Gallurese	1.19	31.83*	0.55	1.01	27.12* °	0.65	0.32	8.68* °	0.64	0.22	5.98* °	1.06	0.15	4.07	1.07	0.2	5.42	2.26
Logudorese	1.21	32.42*	0.27	1	26.91* °	0.33	0.29	7.68* °	0.27	0.14	3.82°	0.34	0.16	4.17	0.58	0.16	4.33	0.93
Nuorese	1.19	31.82*	0.58	0.93	25.02*	0.59	0.31	8.33*	0.49	0.15	3.89	0.74	0.19	5.01	1.25	0.13	3.54	1.82
Sassarese	1.21	32.52*	0.28	0.99	26.47*	0.32	0.26	6.96	0.27	0.14	3.7	0.39	0.14	3.72	0.59	0.2	5.48°	1.18
Italy	0.98	26.25°	0.43	0.8	21.49°	0.63	0.24	6.42	0.63	0.13	3.45	1.01	0.18	4.9	1.51	0.16	4.4	2.07

*T test p-value<0.05 comparing to Italy,

° T test p-value<0.05 comparing to Alghero.

To detect possible footprints of positive selection, the decay of standardized EHH (namely, iHS [23]) has been estimated. Nine genomic regions, harboring more than 200 different genes, showed a signal of positive selection (Table 5). Genomic regions under positive selection on chromosome 9 (from 70,303,655 to 70,400,714 bp) and chromosome 19 (from 22,561,972 to 22,586,080 and from 32,961,206 to 33,175,723) are here described for the first time. Not unexpectedly, a very large chromosomal region showing evidence of positive selection, was found on chromosome 6 (6p21.3), encompassing the human leukocyte antigen (*HLA*) system. Another interesting region is 11q12.1, which contains the 24 genes related to the olfactory receptor activity.

**Table 5:** Nine genomic regions showing signals of positive selection in the Sardinian's genome ordered by |iHS|.

Position NCBI36/hg18	SIZE	n SNP |iHS|>4	n SNP	MAX|iHS|	MAX iHS SNP	*p-value*	empirical *p-value*	genes
chr19: 32,961,206–33,175,723	215	12	69	6.25	rs17714275	3.87 e^−34^	<0.0001	*LINC00662*
chr6: 29,555,703–33,009,633	3454	35	4884	5.37	rs397081	1.64 e^−21^	<0.0001	*GABRB1;MOG; HLA-F;HLA-G; etc;*
chr2: 238,113,451–238,164,950	51	7	37	−5.33	rs2292871	6.62 e^−22^	<0.0001	*MLPH;PRLH;RAB17*
chr9: 70,303,655–70,400,714	97	8	37	5.06	rs11143002	4.86 e^−25^	<0.0001	*PGM5; TMEM252*
chr19: 22,561,972–22,586,080	24	8	16	−4.66	rs4932781	4.49 e^−29^	<0.0001	*LOC440518;LOC100996349*
chr5: 109,659,513–109,731,650	72	13	28	−4.65	rs10478008	9.03 e^−44^	<0.0001	*NA*
chr1: 247,047,666–247,088,866	41	7	15	−4.48	rs12058711	1.05 e^−25^	<0.0001	*SH3BP5L*
chr4: 34,062,734–34,244,104	181	13	46	−4.39	rs11936559	5.37 e^−40^	<0.0001	*NA*
chr11: 55,732,908–56,414,929	682	10	228	4.27	rs12576240	4.79 e^−25^	<0.0001	*OR5T2;OR5T3;OR5T1;OR8H1; etc;*

Column headers: Position on NCBI36/hg18 of region showing evidence for selection; Size in Kb of the genomic region; nSNP |iHS|>4 indicates the number of SNPs with an absolute |iHS| higher than 4 in each region; nSNP is the number of SNPs in each region; Max iHS is the highest value of each region; Max iHS SNP is the polymorphism with the highest value for each region; P-values: nominal p-values; Empirical p-values: after permutation-based multiple testing corrections; Genes: the genes within the region. When, in the genomic region, there are more than 4 genes, only the first 4 are indicated.

## Discussion

A large number of genetic markers belonging to different categories have been employed so far to describe the genetic peculiarity of Sardinians in comparison with other Mediterranean and European populations: classical genetic markers [2], [26], [27], [28], [29], *HLA* system polymorphisms [30], [31], [32], autosomal markers [1], [5], [6], [33], [34], rare cystic fibrosis mutations distribution [35], mitochondrial DNA (mtDNA) polymorphisms [36], [37], [38], [39], [40], [41],Y-chromosome genetic variants, and sequence data [7], [42], [43], [44], [45], [46], [47].

In general, Sardinia appears characterized by a large internal homogeneity [5], [7], like all isolated populations, even though other investigators suggested the presence of genetically different subpopulations in the island [6], [48]. Recently several genome-wide studies have been performed on Sardinian population taking advantage of the genetic homogeneity of the island using also large cohort of individuals [49], [50], [51], [52], [53].

In the present study, we have reconfirmed the high internal homogeneity of Sardinia using four different methods (PCA, F_st_ distance, inflation factor parameter (λ_GC_) and ancestry estimation).

The lack of a subpopulation structure seems clear from PCA. In fact, the multinomial logistic regression model showed that the first four PCs are not able to predict the linguistic macro-areas. Moreover, the inbreeding corrected F_st_ values were spanning from 9.1×10^−5^ to 1.1×10^−4^, and the λ_GC_ values were all nearly 1, indicating both the lack of population differentiation among different areas, and of genetic stratification within the island. The ancestries estimation also suggested a remarkable degree of similarity for all the sampled Sardinian subjects, at the same time a significant heterogeneity when Sardinians are compared to peninsular Italian subjects.

It is nevertheless worthy to note that some Sardinia sub-regions, such as Ogliastra, are actually formed by isolated villages, each of them with a unique demography. Several studies [6], [48], [54] observed differences of linkage disequilibrium (LD) and population structure among these villages. Unfortunately, the limited number of individuals from Ogliastra in our sample (N = 16) did not allow us to test the hypothesis of genetic substructures at the micro-geographic level. The isolation of population has also left its mark on the Sardinians' DNA. In fact a 2-fold increase in the mean homozygosity compared with Italy, is still detectable. Nevertheless we still found evidence for a significant decrease of genome homozygosity in the area surrounding Alghero, which is the linguistic macro-area with the lowest signature of isolation in Sardinia.

We focused on RoHs for a more detailed study on the demographic history of the island. RoHs are regions of the genome in which the inherited copies from both parents are identical as both parents inherited them from a common ancestor at some point in the past (identical by descendent tracts). RoHs are observed in the genome of each individual, and their length is related with their time of origin. RoHs describe different aspects of a population, such as consanguinity, endogamy and demographic events such as bottlenecks. We therefore evaluated the average percentage of the genome covered by RoH>0.5 Mb and RoH>5 Mb (F_RoH0.5_% and F_RoH5_%, respectively) within each Sardinian sub-population compared with those from the Italian peninsula. The F_RoH0.5_% describes the global trend of homozygosity within the sub-populations, while the F_RoH5_% provides information on other phenomena, such as endogamy or recent inbreeding. The average F_RoH0.5_% and the mean sum of the lengths of these segments in Sardinia were higher as compared to Italy (mean sum of F_RoH0.5_ for Sardinia from 72.77 to 82.55 Mb, for Italy 67.55 Mb). These observations are consistent with an ancestral small effective population size (N_e_) in Sardinia and a deeper level of shared ancestry. Once again the Alghero area contrast with those observations showing a F_RoH0.5_% similar to peninsular Italy.

However, we were not able to observe a similar trend for F_RoH5_%, for any of 6 macro-areas. To achieve a deeper detail, we ranked RoHs in six different classes. On average, in Sardinia the mean sum of the shortest RoHs (0.5–1 Mb and 1–2 Mb) was significantly longer than in Italy. This phenomenon can be explained as the result of common extended haplotypes probably inherited from both parents, that are frequent in isolates and small communities [55].

Other macro-area such as Campidanese and Gallura (concerning RoH from 2 to 8 Mb) and Logudorese and Nuorese (RoH 2–4 Mb) still retain traces of endogamy when compared to peninsular Italy.

Again, in the Alghero area, RoHs above the threshold of 2 Mb, were shorter and less common than in the other Sardinian populations; this finding indicates significant lower endogamy and consanguinity degree in this subpopulation. It should be noted that the North-Western town of Alghero is a Catalan-speaking community and this language is a remarkable exception from all Sardinian varieties of dialects. The Alghero's dialect derives from historical events which affected the city in the Middle Ages when the population was swelled by the arrival of Catalan-speaking colonists [56].

In our knowledge, only one study has previously assessed genome-wide patterns of homozygosity in the Sardinian population [5]. Although the criteria used for the identification of RoHs are slightly different between the present study and that of Pardo and colleagues, the results of the two studies are consistent.

Additionally, we searched for footprints of positive selection in the Sardinian genome by using extended haplotype homozygosity and iHS test. Our results identified some genomic regions not previously described as being under positive selection, that may be considered as novel candidates worthy of investigation for positive selection in Sardinian population. Among them, the *TMEM252* gene (ID 169693) and *PGM5* gene (ID 5239) region, and a region on chromosome 19 containing a long non-coding RNA (LINC00662). As expected, we re-captured many of the previously described signals of recent positive selection. Specifically, the *PRLH* gene (ID 51052) and *MLPH* gene (ID 79083), both located on the long arm of chromosome 2, which are under selection in Mideast and European populations [57], the *SH3BP5L* gene (ID 80851) [58], and a region on chromosome 11 containing several olfactory-related genes [59]. As reported in literature, the region of the human leukocyte antigen (*HLA*) system is under positive selection in the Europeans, Mideast and South Asian populations [57]. In our study we did not find the lactase gene (*LCT* ID 3938) among the regions under positive selection, as reported also by other studies [57], [60].

## Conclusion

Although the main limitation of our study is that the information on Sardinian individuals' origins were based only on their birth place, our study reconfirmed by using different approaches the high degree of internal genetic homogeneity in Sardinia. We have shown that the genome of the Sardinians has mean inbreeding coefficients which are higher than those of mainland Italians. Furthermore, the Sardinian's genome still preserves traces of the elaborate demographic history of the island. Between the macro-areas analyzed, the area surrounding Alghero shows less inbreeding than others, according to its peculiar history and underlined also by the local dialect. Several genomic regions showing signals of positive selection were identified, some of them not previously described and as such worthy of further investigation. In the near future, our results could be confirmed by re-sequencing the genes/regions showing signature of positive selection and by identifying potentially functional SNPs/haplotypes.

## Supporting Information

Figure S1 **Box plot distribution of the first four eigenvectors in the 6 macro-areas.**
(JPG)

Text S1 The R code used to compute 1) the Hudson estimator [17], as suggested in Bhatia *et al.* [18]. 2) inbreeding corrected F_st_ estimator as suggested in Reich *et al.* [16].
(DOC)
