# Supplementary figures and images for: Sardinians Genetic Background Explained by Runs of Homozygosity and Genomic Regions under Positive Selection
Source: PLoS One. 2014 Mar 20;9(3):e91237. doi: 10.1371/journal.pone.0091237 (PMC3961211; doi:10.1371/journal.pone.0091237)

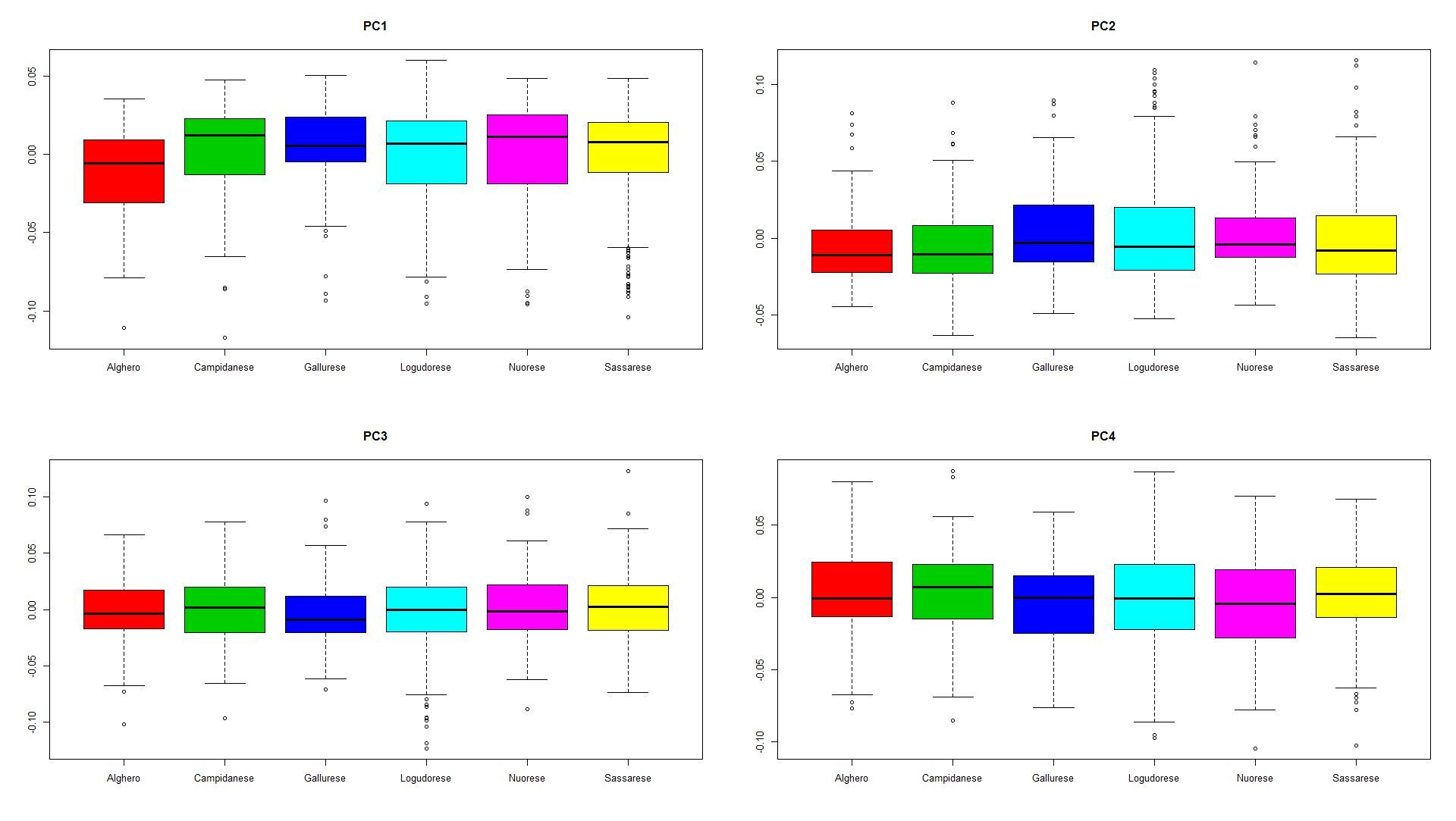

Supplement: Figure S1 — Box plot distribution of the first four eigenvectors in the 6 macro-areas. (JPG) [file pone.0091237.s001.jpg]
