# Supplementary material for: Sardinians Genetic Background Explained by Runs of Homozygosity and Genomic Regions under Positive Selection
Source: PLoS One. 2014 Mar 20;9(3):e91237. doi: 10.1371/journal.pone.0091237 (PMC3961211; doi:10.1371/journal.pone.0091237)
Supplement: Text S1 — The R code used to compute 1) the Hudson estimator [17], as suggested in Bhatia et al. [18]. 2) inbreeding corrected Fst estimator as suggested in Reich et al. [16]. (DOC) [file pone.0091237.s002.doc]

**R code to compute Hudson Fst estimator**

# input data frame pop1 is a N x 4 matrix

# where N is the number of SNPs

# row names correspond to the SNP name

# MAF represent the minor allele frequency

# NCHROBS represent the number of chromosome observed (2 x sample size)

# A1 common allele

# A2 variant allele

# example

> head(pop1,5)

A1 A2 MAF NCHROBS

rs3094315 G A 0.18590 156

rs3131972 A G 0.18350 158

rs3115860 C A 0.13160 152

rs12562034 A G 0.09615 156

rs12124819 G A 0.20950 148

rs2980300 A G 0.13290 158

# similarly for pop2

Hudson.Fst <- function(pop1,pop2,call.rate = 0.95,top.number = 10){

# remove the SNPs that are not in common between the 2 populations

snp.to.keep <- intersect(row.names(pop1),row.names(pop2))

if (length(snp.to.keep) == 0){print("Error: no SNP in common");return(NULL)}

pop1.k <- pop1[snp.to.keep,]

pop2.k <- pop2[snp.to.keep,]

# change the reference allele if is not concordant between the 2 populations

if (sum(pop1.k$A1 == pop2.k$A1) != length(snp.to.keep)){

idx <- which(pop1.k$A1 != pop2.k$A1)

idx.rev <- which(pop1.k$A1 != pop2.k$A1 & pop1.k$A1 == pop2.k$A2)

idx.rm <- which(pop1.k$A1 != pop2.k$A1 & pop1.k$A1 != pop2.k$A2)

if(length(idx.rev) > 0){

provv <- pop1.k$A1[idx.rev]

pop1.k$A1[idx.rev] <- pop1.k$A2[idx.rev]

pop1.k$A2[idx.rev] <- provv

pop1.k$MAF[idx.rev] <- 1 - pop1.k$MAF[idx.rev]

}

if(length(idx.rm) > 0){

pop1.k <- pop1.k[-idx.rm,]

pop2.k <- pop2.k[-idx.rm,]}}

# remove SNPs with low call rate in one or both populations

N1 <- pop1.k$NCHROBS

N2 <- pop1.k$NCHROBS

idx.rm.pop1 <- which(N1 < max(N1)*call.rate)

idx.rm.pop2 <- which(N2 < max(N2)*call.rate)

idx.rm.all <- union(idx.rm.pop1,idx.rm.pop2)

pop1.k <- pop1.k[-idx.rm.all,]

pop2.k <- pop2.k[-idx.rm.all,]

# compute Hudson SNP_Fst and global Fst estimators

p1 <- pop1.k$MAF

p2 <- pop2.k$MAF

n1 <- pop1.k$NCHROBS

n2 <- pop2.k$NCHROBS

fst.N <- (p1 - p2)^2 - p1*(1-p1)/(n1-1) - p2*(1-p2)/(n2-1)

fst.D <- p1*(1-p2) + p2*(1-p1)

Fst.v <- fst.N/fst.D

names(Fst.v) <- row.names(pop1.k[-idx.rm.all,])

Fst.o <- Fst.v[order(Fst.v,decreasing=TRUE)]

mu1 <- mean(fst.N)

mu2 <- mean(fst.D)

se1 <- sd(fst.N)/sqrt(length(fst.N))

se2 <- sd(fst.D)/sqrt(length(fst.D))

F.global <- mu1/mu2

se.F <- sqrt(se1^2+se2^2)

F_L95 <- F.global - 1.96*se.F

F_U95 <- F.global + 1.96*se.F

Z <- F.global/se.F

p <- 2*(1 - pnorm(Z))

if(p < 2e-16){p <- "less than 2e-16"}

output <- list()

output[[1]] <- c(F.global,F_L95,F_U95,p)

names(output[[1]]) <- c("Hudson.Fst","L.95%.CI","U.95%.CI","p.val")

output[[2]] <- data.frame(Fst.o[1:top.number])

names(output[[2]]) <- c("Hudson.Fst")

return(output)}

**R code to compute Reich Fst estimator (inbreeding corrected Fst)**

# input data frame pop1 is a N x 5 matrix

# where N is the number of SNPs

# row names correspond to the SNP name

# x0 represent the number of samples with 0 copies of the variant allele

# x1 represent the number of samples with 1 copy of the variant allele

# x2 represent the number of samples with 2 copies of the variant allele

# A1 common allele

# A2 variant allele

# example

> head(pop1,5)

x0 x1 x2 A1 A2

rs28659788 80 18 0 G C

rs3094315 68 29 1 G A

rs3131972 68 29 1 A G

rs3115860 80 13 0 C A

rs3131969 82 15 1 A G

# similarly for pop2

Reich.Fst <- function(pop1,pop2,call.rate = 0.95, top.number = 10){

# remove the SNPs that are not in common between the 2 populations

snp.to.keep <- intersect(row.names(pop1),row.names(pop2))

if (length(snp.to.keep) == 0){print("Error: no SNP in common");return(NULL)}

pop1.k <- pop1[snp.to.keep,]

pop2.k <- pop2[snp.to.keep,]

# change the reference allele if is not concordant between the 2 populations

if (sum(pop1.k$A1 == pop2.k$A1) != length(snp.to.keep)){

idx <- which(pop1.k$A1 != pop2.k$A1)

idx.rev <- which(pop1.k$A1 != pop2.k$A1 & pop1.k$A1 == pop2.k$A2)

idx.rm <- which(pop1.k$A1 != pop2.k$A1 & pop1.k$A1 != pop2.k$A2)

if(length(idx.rev) > 0){

provv <- pop1.k$A1[idx.rev]

pop1.k$A1[idx.rev] <- pop1.k$A2[idx.rev]

pop1.k$A2[idx.rev] <- provv

provv <- pop1.k$x0[idx.rev]

pop1.k$x0[idx.rev] <- pop1.k$x2[idx.rev]

pop1.k$x2[idx.rev] <- provv}

if(length(idx.rm) > 0){

pop1.k <- pop1.k[-idx.rm,]

pop2.k <- pop2.k[-idx.rm,]}}

# remove SNPs with low call rate in one or both populations

x0 <- pop1.k$x0

x1 <- pop1.k$x1

x2 <- pop1.k$x2

s <- x0 + x1 + x2

y0 <- pop2.k$x0

y1 <- pop2.k$x1

y2 <- pop2.k$x2

t <- y0 + y1 + y2

idx.rm.pop1 <- which(s < max(s)*call.rate)

idx.rm.pop2 <- which(t < max(t)*call.rate)

idx.rm.all <- union(idx.rm.pop1,idx.rm.pop2)

x0 <- x0[-idx.rm.all]

x1 <- x1[-idx.rm.all]

x2 <- x2[-idx.rm.all]

s <- s[-idx.rm.all]

y0 <- y0[-idx.rm.all]

y1 <- y1[-idx.rm.all]

y2 <- y2[-idx.rm.all]

t <- t[-idx.rm.all]

# compute SNP_Fst and global Fst estimators in presence of inbreeding

e.x <- ((x1 + 2*x2)/(2*s) - (y1 + 2*y2)/(2*t))^2 + x1/(4*s*s) + y1/(4*t*t)

e.h1 <- (x0*x2 + (x0 + x2)*x1/2 + x1*(x1-1)/4)/(s*(s-1))

e.h2 <- (y0*y2 + (y0 + y2)*y1/2 + y1*(y1-1)/4)/(t*(t-1))

N <- e.x - e.h1/s - e.h2/t

D <- N + e.h1 + e.h2

Fst.v <- N/D

names(Fst.v) <- row.names(pop1.k[-idx.rm.all,])

Fst.o <- Fst.v[order(Fst.v,decreasing=TRUE)]

F.global <- sum(N)/sum(D)

se1 <- sd(N)/sqrt(length(N))

se2 <- sd(D)/sqrt(length(N))

se.F <- sqrt(se1*se1 + se2*se2)

F_L95 <- F.global - 1.96*se.F

F_U95 <- F.global + 1.96*se.F

Z <- F.global/se.F

p <- 2*(1 - pnorm(Z))

if(p < 2e-16){p <- "less than 2e-16"}

output <- list()

output[[1]] <- c(F.global,F_L95,F_U95,p)

names(output[[1]]) <- c("Reich.Fst","L.95%.CI","U.95%.CI","p.val")

output[[2]] <- data.frame(Fst.o[1:top.number])

names(output[[2]]) <- c("Reich.Fst")

return(output)}
